# FcγRIIIa Expression on Monocytes in Rheumatoid Arthritis: Role in Immune-Complex Stimulated TNF Production and Non-Response to Methotrexate Therapy

**DOI:** 10.1371/journal.pone.0028918

**Published:** 2012-01-03

**Authors:** Dawn L. Cooper, Stephen G. Martin, James I. Robinson, Sarah L. Mackie, Christopher J. Charles, Jackie Nam, YEAR Consortium, John D. Isaacs, Paul Emery, Ann W. Morgan

**Affiliations:** 1 NIHR-Leeds Musculoskeletal Biomedical Research Unit, University of Leeds, Leeds, United Kingdom; 2 YEAR Consortium; 3 Institute of Cellular Medicine, Newcastle University, Newcastle upon Tyne, United Kingdom; University of Leuven, Rega Institute, Belgium

## Abstract

**Objective:**

The expression of FcγRIIIa/CD16 may render monocytes targets for activation by IgG-containing immune complexes (IC). We investigated whether FcγRIIIa/CD16 was upregulated in rheumatoid arthritis (RA), associated with TNF production in response to IC-stimulation, and if this predicted response to methotrexate therapy.

**Methods:**

FcγRIIIa/CD16 expression on CD14^low^ and CD14^++^ monocytes was measured by flow cytometry in healthy controls and RA patients (early and long-standing disease). Intracellular TNF-staining was carried out after *in vitro* LPS or heat-aggregated immunoglobulin (HAG) activation. FcγRIIIa/CD16 expression pre- and post-steroid/methotrexate treatment was examined.

**Results:**

Increased FcγRIIIa/CD16 expression on CD14^++^ monocytes in long-standing RA patients compared to controls was demonstrated (p = 0.002) with intermediate levels in early-RA patients. HAG-induced TNF-production in RA patients was correlated with the percentage of CD14^++^ monocytes expressing FcγRIIIa/CD16 (p<0.001). The percentage of CD14^++^ monocytes expressing FcγRIIIa/CD16 at baseline in early DMARD-naïve RA patients was negatively correlated with DAS28-ESR improvement 14-weeks post-methotrexate therapy (p = 0.003) and was significantly increased in EULAR non-responders compared to moderate (p = 0.01) or good responders (p = 0.003). FcγRIIIa/CD16 expression was not correlated with age, presence of systemic inflammation or autoantibody titers.

**Conclusion:**

Increased FcγRIIIa/CD16 expression on CD14^++^ monocytes in RA may result in a cell that has increased responsiveness to IC-stimulation. This monocyte subset may contribute to non-response to methotrexate therapy.

## Introduction

IgG-containing immune complexes (IC), such as those containing rheumatoid factors (RFs) and cyclic citrullinated peptide (CCP) autoantibodies, are found abundantly in serum and synovial fluid of patients with rheumatoid arthritis (RA) [1], [2]. ICs activate various cell types following Fcγ receptor (FcγR) and complement receptor binding and lead to a diverse range of effector functions. FcγRs play important roles in the initiation and regulation of many immunological processes [3]–[5]. The importance of an appropriate balance between activating and inhibitory FcγRs in the regulation of animal models of arthritis is well recognised [6], [7]. A dominant role for FcγRIIIa in IgG IC-mediated inflammatory responses and in type I, II and III hypersensitivity reactions has been highlighted in diverse animal models [8], including autoantibody-induced arthritis [9]. FcγRIIIa knockout mice are protected from IC-induced arthritis [10], [11] with FcγRIIIa-mediated mechanisms, but not complement, dominating in promoting organ-specific destructive pathologies [12], [13]. We have recently demonstrated that genetic variation in *FCGR3A* is a risk factor for the development of autoantibody-positive RA [14].

Cells of the monocyte/macrophage lineage play important roles in RA pathogenesis, particularly the perpetuation of inflammation, and are potential targets for activation by ICs. Activated macrophages are the predominant infiltrating cell type found in rheumatoid synovium, pannus and nodules [15], [16]. FcγR cross-linking on macrophages potentially initiates phagocytosis, antigen presentation, antibody-dependant cell-mediated cytotoxicity (ADCC) and release of pro-inflammatory cytokines and tissue destructive mediators [17]. The migration of monocytes from blood to synovial tissue and their differentiation into macrophages may be an important step in disease pathogenesis [18]. Macrophages are the major source of pro-inflammatory cytokines and chemokines in the inflamed RA joint, including tumour necrosis factor (TNF), interleukin-1 (IL-1), interleukin-8 (IL-8) and granulocyte-macrophage colony-stimulating factor (GM-CSF) [15], [19]. FcγRIIIa cross-linking has been specifically implicated in cytokine release from adherent human monocytes/macrophages [20], [21]. These cytokines are intimately involved in the disease process as demonstrated by the clinical efficacy of TNF or IL-1 blockade in RA [22], [23]. Osteoclasts, multinucleated giant cells with the capacity to resorb bone, are also derived from a blood-borne monocyte precursor and have been implicated in the destructive disease process [24].

In human peripheral blood, monocyte subpopulations with distinct functional properties have been defined by their expression of CD14 and CD16 (FcγRIIIa) [25], [26]. Monocyte subsets were initially defined as CD14^low^/CD16^++^ and CD14^++^/CD16^neg/low^ following work in healthy control subjects [27]. The CD14^low^ subpopulation accounts for approximately 7–10% of circulating monocytes in healthy individuals. Recent studies have confirmed that this is a distinct monocyte subpopulation resembling the murine “patrolling” Gr1^-^ monocytes, which appear to play a role in immunosurveillance and the release of proinflammatory cytokines, including TNF, in response to virally infected cells and nucleic acids [26]. Previous studies have shown that the numbers of CD14^low^ monocytes are expanded during infectious and inflammatory conditions, such as RA [28]–[31], however, this finding has not been confirmed in all studies [32]. In contrast, the “classical” or “inflammatory” CD14^++^ monocyte subpopulation that resembles murine Gr1^+^ monocytes was previously believed to act as a phagocytic scavenger cell, removing apoptotic cells and aiding in the resolution of inflammation [33]. More recent studies have demonstrated reactive oxygen species production and cytokine release in response to lipopolysaccharide (LPS) [26]. This cellular subset does not express FcγRIIIa/CD16 under normal conditions; however, levels are variable and can be increased in inflammatory disease [6], [34]–[37]. In RA, interest has previously focussed on the CD14^low^ subset due to the high expression of FcγRIIIa/CD16 and its ability to release TNF upon activation [38]. However, these findings derive from observations on healthy control cells and FcγR-independent activators, such as LPS [38]. The induction of proinflammatory cytokine release via ICs cross-linking FcγRIIIa/CD16 on monocytes/macrophages is a possible additional mechanism for cellular activation in RA [39]. Various studies have demonstrated that IC, such as anti-CCP/ACPA-containing IC [36], [40] and collagen-containing IC [41], are able to induce TNF production from monocytes/macrophages. This has been demonstrated to be in a dose-dependent manner [40]. Furthermore IC taken from serum and synovial fluid of RA patients can induce TNF cytokine release from these cells [42]. It remains unknown whether CD14^++^ monocytes can produce substantially increased IC-stimulated TNF under inflammatory conditions when FcγRIIIa/CD16 expression is up regulated. In addition, genetic polymorphism within *FCGR3A* whereby a valine to phenylalanine substitution occurs (*FCGR3A-*158F/V) has been shown to influence the affinity of the receptor for IgG. This may also modulate activation through FcγRIIIa/CD16.

We investigated whether monocytes from RA patients expressed higher levels of FcγRIIIa/CD16 resulting in a cell that is potentially more sensitive to intravascular IC stimulation. Furthermore, we explored whether upregulated FcγRIIIa/CD16 expression was associated with modulation of monocyte functions by assessing cytokine production following both LPS and IC activation. We additionally hypothesised that upregulation of FcγRIIIa/CD16 in RA may be associated with a reduced response to DMARD therapy, due to a persistent inflammatory drive. Methotrexate remains the initial treatment of choice in RA, but is widely recognised to fail to control disease activity in a sizeable proportion of patients. The potential clinical significance of this biological pathway was therefore evaluated in a cohort of DMARD-naïve RA patients receiving methotrexate as first-line therapy.

## Materials and Methods

### Patients and controls

Adult RA patients (n = 38, mean age = 52 [range 27–80], 22 female) with long-standing disease (>2 years) who were taking concurrent disease modifying anti-rheumatic drugs (DMARDs) were recruited from rheumatology clinics in Leeds. Patients fulfilled the 1987 American College of Rheumatology classification criteria [43]. DMARD-naïve early RA patients (n = 73, mean age = 56 (range 29–78), 21 female) were recruited from the Yorkshire Early Arthritis Register (symptom duration <1 year). Forty two patients were reviewed following methotrexate therapy (10 mg weekly methotrexate, increasing stepwise to 20 mg by week 4) with an initial bolus of systemic steroids (25 0mg IV Methylprednisolone) for symptom control at initiation. Peripheral blood samples were taken at baseline and following 2 and 14-weeks of therapy. Blood samples were simultaneously obtained for determination of CRP, ESR, anti-CCP and RF titres by routine clinical laboratory methods. Four-variable DAS28-ESR scores were determined at baseline and 14-weeks post-initiation of therapy and used to define patient responses according to EULAR response criteria (good, moderate or non-response). The presence or absence of hand and foot erosions on X-ray at baseline was also assessed. Healthy controls (n = 41, mean age = 44 (range 28–72), 18 female) were not experiencing any auto-inflammatory or infectious events at time of blood donation. Additional elderly control subjects with no concurrent inflammatory or infectious diseases were recruited from either general rheumatology or ophthalmology clinics (n = 21, mean age = 74 (range 55–90)). The participants gave informed written consent in accordance with the Declaration of Helsinki, and the study was approved by the National Research Ethics Service Committee Yorkshire & The Humber - Leeds East.

### Flow cytometry

**Figure 1:**
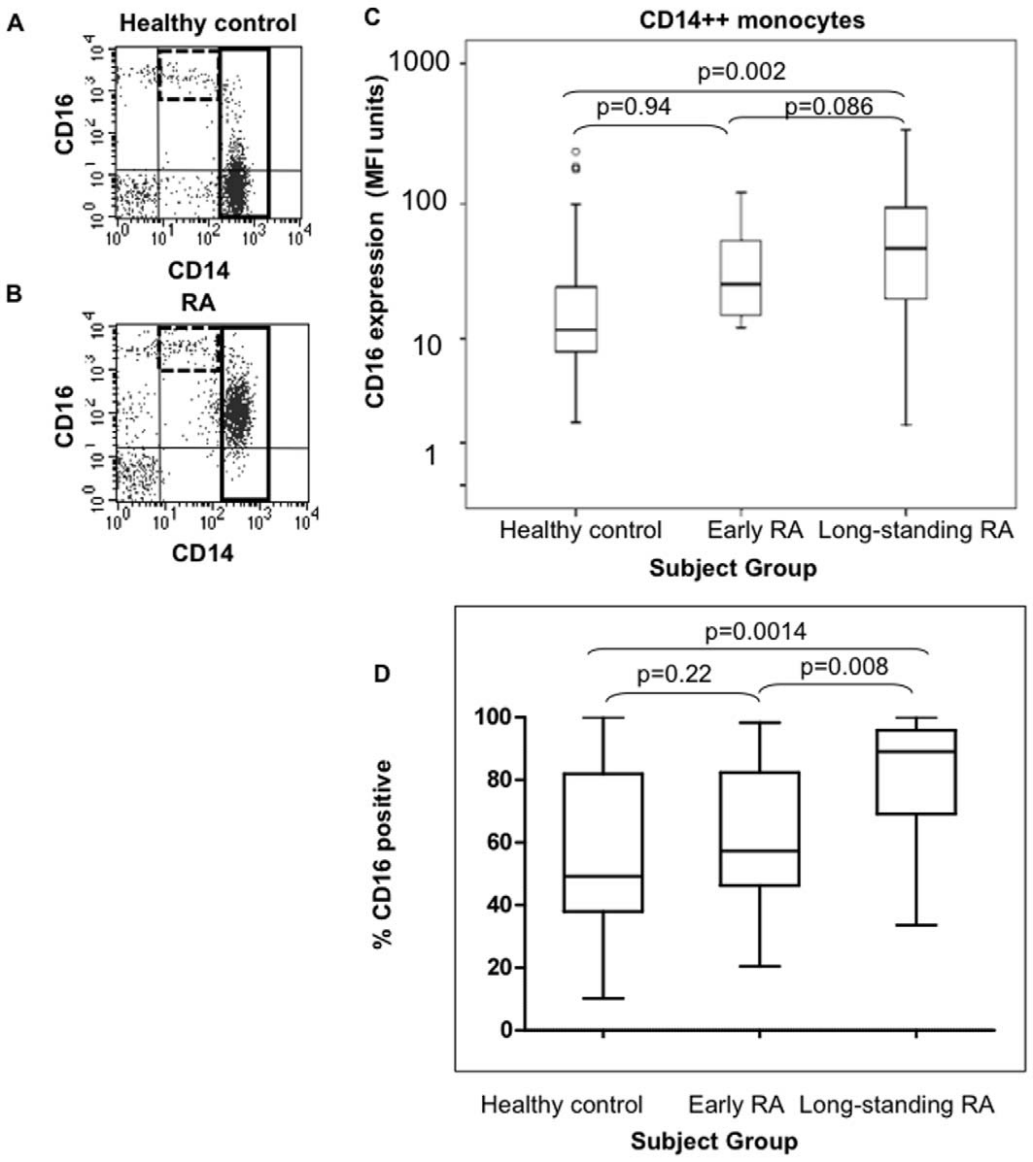
CD16 expression on peripheral blood monocytes in healthy control and RA patients. Example flow cytometric analysis of peripheral blood monocytes from a single healthy control subject (A) and a long-standing RA patient (B). Monocytes were initially gated based on light forward and side scatter (gated on monocytes and the upper portion of lymphocytes). Within this population, monocyte subsets were distinguished by their staining with antibodies to CD14 and CD16. Examples of the separate gates created around the CD14^low^/CD16^++^ populations (dashed boxes) and the CD14^++^/CD16^neg/low^ population (solid boxes) are shown. The same samples stained with anti-CD14-PerCP and IgG1-phycoerythrin (PE) isotype control in place of anti-CD16-PE were used to set gates. CD14^neg^/CD16^++^ cells were excluded from the CD14^low/^CD16^++^ based on gates set using staining with IgG2a-PerCP isotype control. (C) CD16 expression levels (MFI units) on CD14^++^ monocytes in control (n = 40), early RA (n = 42) and long-standing RA patients (n = 38). (D) Percentage of CD14^++^ monocytes expressing CD16 in the same healthy control, early RA and long-standing RA patients. Box plots represent median and upper and lower quartiles and dots represent outliers. Statistics; Mann-Whitney U test.

NH_4_Cl-lysed heparinised blood samples were incubated with 2 µg/ml normal human immunoglobulin (Novartis, Hanover, Germany), to block non-specific binding for 30 min on ice. This immunoglobulin concentration was chosen based on the maximal human immunoglobulin concentration able to prevent non-specific binding of IgG isotype control (Fig. S1A) while preserving specific staining with anti-CD16 antibody (Fig. S1B). In 5 RA patients and 3 healthy controls this immunoglobulin concentration was demonstrated to have no detrimental effect on the CD16-PE staining (Fig. S1C, with or without immunoglobulin p = 0.666). Cells were stained (30 min, ice) with anti-CD16-PE (clone 3G8, Caltag-Medsystems, Buckingham, UK) and anti-CD14-PerCP (clone MφP9, BD Biosciences, Oxford, UK) or irrelevant isotype controls. Cells were washed in phosphate buffered Saline containing 0.5% bovine serum albumin and flow cytometry data collected on FACSCalibur (Becton Dickinson, Oxford, UK). Monocyte subsets were identified on the basis of forward scatter/side scatter characteristics (FSC/SSC) and expression of CD14. Separate gates were created around CD14^low^/CD16^++^ and CD14^++^/CD16^neg/low^ monocytes (Fig. 1A; dashed and solid boxes respectively). The CD16 geometric mean fluorescence intensity (MFI) and the percentage of CD16 positive cells were determined.

### Analysis of TNF production

Thawed cryopreserved peripheral blood mononuclear cells (PBMC) were incubated for 1 hour at 37°C with 1 µg/ml LPS (Sigma-Aldrich, Dorset, UK) or 100 µg/ml heat-aggregated immunoglobulin (HAG; soluble immunoglobulin (Novartis, Hanover, Germany) incubated for 1 hour, 63°C) prior to addition of a Monensin protein transport inhibitor (Golgistop™, BD Biosciences, Oxford, UK) and further incubation for 3 hours at 37°C in 5% CO_2_. Cells were washed and incubated with anti-CD14-PerCP (30 min, 4°C in the dark) followed by washing and incubation with Leucoperm™ fixing reagent A (Serotec, Oxford, UK) (15 minutes, room temperature in the dark). The cells were washed and incubated with anti-TNF-PE or IgG1-PE (Serotec, Oxford, UK) in the presence of Leucoperm™ permeabilisation buffer B (30 min, room temperature in the dark) before washing and resuspending in PBS-BSA for immediate analysis (Becton Dickinson LSRII). Monocytes were identified by FSC/SSC and CD14 expression. The geometric MFI of TNF staining was determined for all CD14-positive monocytes.

### Genotyping

EDTA blood samples were frozen for DNA extraction and genotyping. *FCGR3A* genotyping (FCGR3A-158F/V) was carried out by direct sequencing of the PCR product [44], [45].

### Statistical Analysis

Data are summarised in box-plot diagrams (median and interquartile ranges (IQR)) and compared using the Mann-Whitney U test. Spearman's test was used for correlations (p values <0.05 statistically significant). Wilcoxon signed-ranks test was used for paired data samples. Potential differences in baseline characteristics for EULAR non-responders compared with moderate or good EULAR responders by week 14 were examined to identify baseline characteristics that might influence response. Analysis was performed using SPSS 15.0 for windows (Chicago, Illinois, USA).

## Results

### Monocyte CD16 expression in healthy controls and RA patients

**Figure 2:**
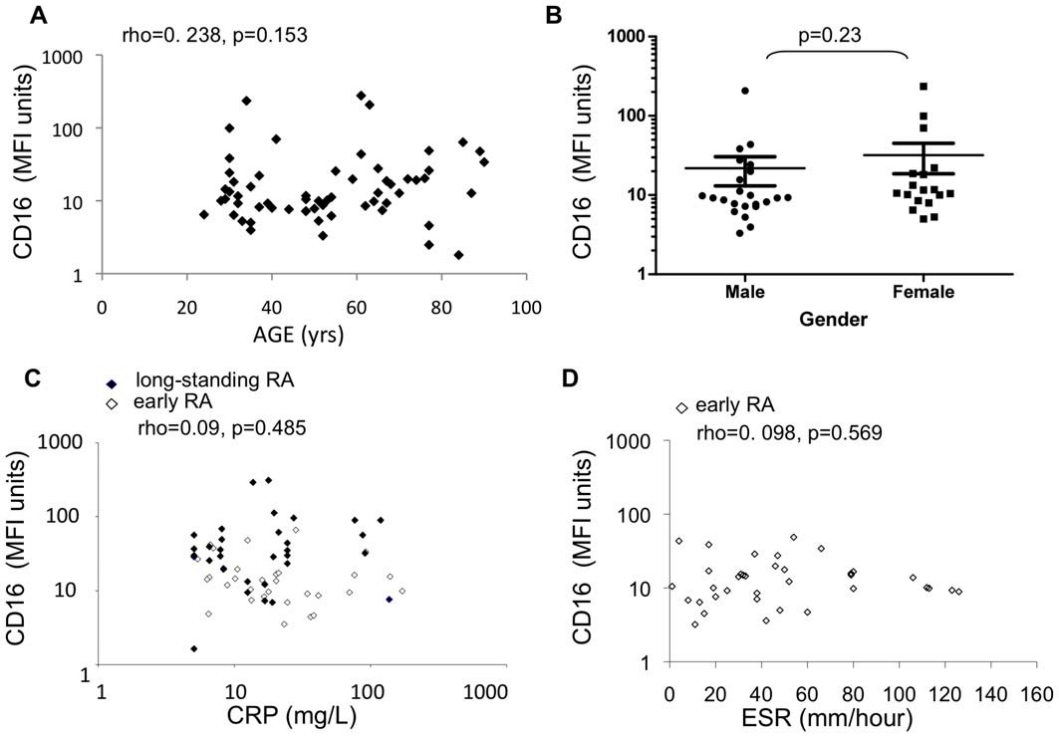
Correlation of age, gender, CRP and ESR with FcγRIIIa/CD16 expression on CD14^++^ monocytes. (A) Healthy control subjects across a range of ages (x-axis) were tested for CD16 expression levels (MFI units, y-axis) on the CD14^++^ monocyte subset. (B) Healthy control subjects were stratified according to gender and levels of CD16 expression examined. (C) CRP levels in long-standing RA patients (closed points) and early RA patients (open points) were tested at the time of donating blood sample and examined for correlation with CD16 expression levels (MFI units, y-axis). (D) ESR values (x-axis) were measured in early RA patients only and examined for correlation with CD16 expression level (MFI units, y-axis). Statistics; Spearman's correlation.

Peripheral blood leukocytes from long-standing RA patients, early-RA patients and unrelated healthy controls were examined for CD16 expression on CD14^low^ and CD14^++^ monocytes using flow cytometry (Fig. 1). The percentage of CD14^low^/CD16^++^ monocytes among total CD14-positive cells in long-standing RA patients (median 7.98% (IQR 3.6 to 9.6), n = 38) was not significantly different from control subjects (median 6.51% (IQR 4.4 to 10.4), n = 40) (p = 0.305; Mann-Whitney U). There was no significant difference in CD16 expression (MFI units) on CD14^low^/CD16^++^ monocytes between healthy controls (median 927 (IQR 578-999)) and long-standing RA patients (median 1127 (IQR 953-1581), (p = 0.299)). More strikingly, the CD14^++^/CD16^neg/low^ subset expressed variable levels of CD16; most CD14^++^ monocytes in healthy control subjects were negative/low for CD16 expression (Fig. 1A). In RA patients, positive staining for CD16 was shown on this subset (Fig. 1B; high CD16-expressing patient). Expression levels were significantly higher in patients with long-standing disease than in healthy controls (p = 0.002; Fig. 1C). CD16 expression levels in early-RA were intermediate between healthy control and long-standing RA patients and were not significantly different from either (p = 0.94, p = 0.086, respectively). The percentages of CD14^++^ monocytes expressing CD16 was also higher in patients with long-standing disease than healthy controls (p = 0.0014; Fig. 1D). Early-RA patients were not significantly different than healthy controls in percentages of CD16 positive CD14^++^ monocytes, but were significantly different from long-standing RA patients (p = 0.22 and 0.008 respectively; Fig. 1D). There was no correlation between CD16 expression levels on CD14^++^ monocytes and increasing age in control subjects (p = 0.13, Fig. 2A), no significant difference when control subjects were stratified according to gender (p = 0.23, Fig. 2B). There was no correlation with the level of inflammation (CRP (p = 0.485, Fig. 2C) or ESR (p = 0.569, Fig. 2D)) in early and late RA patients.

### TNF production from CD14-positive monocytes

**Figure 3:**
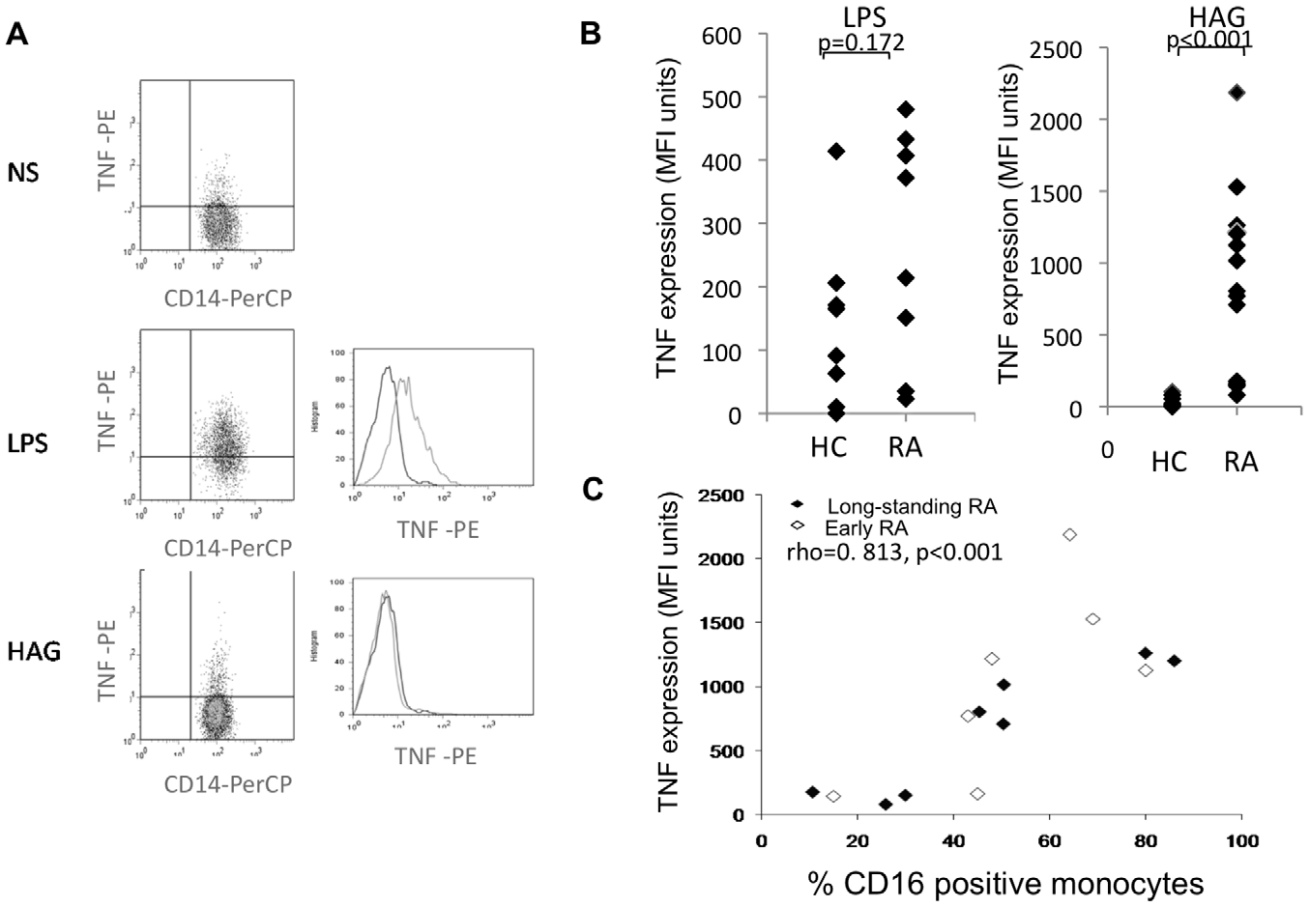
TNF Intracellular staining in CD14 positive monocytes from healthy control and RA patients. A) Thawed A) Cryopreserved PBMC from control and RA patients were stimulated for 4hrs with LPS (1 µg/ml), heat-aggregated Ig [HAG] (100 µg/ml) or the absence of stimulation (NS). Representative dot plots/histograms are shown for all treatments in a HC individual and for HAG stimulation in a RA patient (bottom panel). Intracellular staining was performed using anti-human TNF-PE or isotype control-PE antibodies and MFI units of expression measured. B) Intracellular staining for TNF in response to LPS (left panel) and HAG (right panel) in healthy control (HC, n = 8) and RA (n = 15) subjects. C) Correlation between HAG-stimulated TNF intracellular staining and % CD16 positive monocytes in RA patients. The percentage of CD14^++^ monocytes expressing CD16 (x-axis) and the MFI of anti-TNF-PE expression (y-axis) were determined by flow cytometry. Open diamonds represent early RA patient and shaded diamonds represent long-standing RA patients. Correlation coefficient spearman's rho = 0·813, p<0.001.

TNF production triggered by LPS or IC stimulation (HAG) was measured by intracellular staining of CD14–expressing monocytes (Fig. 3A). LPS stimulation elicited TNF production by CD14-expressing monocytes at a similar level between control (n = 8) and RA (n = 8) cryopreserved PBMC (Fig. 3B). RA monocytes (n = 15) demonstrate greater TNF production on HAG stimulation than controls (p<0.001, Fig. 3B). The extent of HAG-stimulated intracellular TNF staining in RA patients was correlated with the percentage of CD14^++^monocytes expressing CD16 (r = 0.813, p<0.001; Fig. 3C).

### CD16^pos^ expression on CD14^++^ monocytes is predictive of response to methotrexate treatment

**Figure 4:**
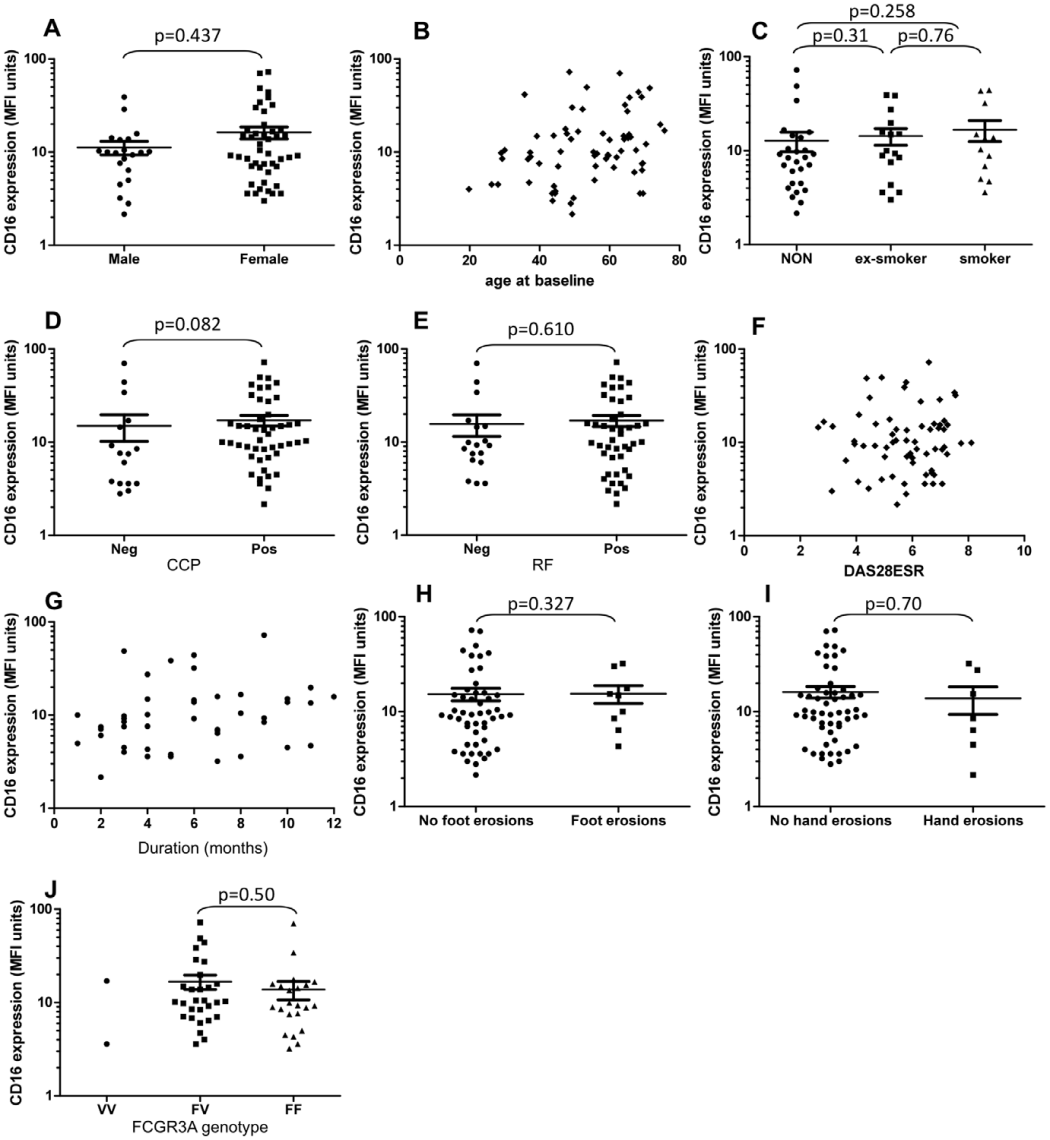
Baseline characteristics of early RA patients prior to methotrexate therapy and correlations with CD16 expression. CD16 expression levels were examined on CD14^++^ monocytes for correlations with baseline characteristics in a cohort of early RA patients prior to methotrexate therapy. Demographics examined were gender (A), age at baseline (B), smoking status (C), CCP autoantibody-positivity (D), RF-positivity (E), baseline DAS28ESR (F), disease duration at initiation of therapy (G), foot erosions (H), hand erosions (I) and *FCGR3A* genotype (J). Relationships between these baseline characteristics and EULAR responses are shown in Table 1.

Clinical predictors of CD16 expression level in RA patients. Patient demographics of a cohort of early RA patients are shown in Table 1. The influence that the main baseline patient characteristics have on CD16 expression levels on CD14++ monocytes are shown in Figure 4. Baseline CD16 expression on CD14++ monocytes was not influenced by age (rho = 0.266, p = 0.057 Fig. 4B), baseline DAS28ESR scores (rho = 0.041; p = 0.743, Fig. 4F) or symptom duration up to one year (rho = 0.262, p = 0.816 Fig. 4G). There was no significant difference in CD16 expression on CD14++ monocytes in early RA patients stratified according to gender (p = 0.437, Fig. 4A), smoking status (current smoker vs. non-smoker p = 0.258, Fig. 4C), the presence of autoantibodies (CCP pos vs. neg p = 0.082, RF pos vs. neg p = 0.610, Fig. 4D and Fig. 4E respectively), or the presence or absence of hand or foot erosions at presentation (p = 0.327, Fig. 4H and p = 0.70, Fig. 4I respectively). Genetic polymorphisms within the *FCGR3A* (*FCGR3A-158F/V*) gene did not influence absolute FcγRIIIa/CD16 expression level in early RA patients (FV vs. FF p = 0.506, Fig. 4J).

**Table 1:** Early RA patient baseline demographics and associations with response to steroid/methotrexate therapy.

	Entire Cohort	NR	MOD/GOOD	NR vs. MOD/GOOD
	n = 73	n = 11	n = 31	*p value*
Gender (% women)	69	91	64	P = 0.03 (‡)
Age (median years)	51	59	48	P = 0.071 (φ)
Smoking (% ever smoked)	45.7	66	38.4	P = 0.245 (‡)
Smoking (% current smokers)	25.7	22.2	26.9	P = 1.0 (‡)
CCP titre (mean)	204	229	194	P = 0.539 (φ)
CCP positive (%)	86	90	84	P = 0.7448 (‡)
RF titre (mean)	145	192	127	P = 0.156 (φ)
RF positive (%)	80	80	80	P = 1.00 (‡)
DAS28ESR baseline	5.6	5.20	5.74	P = 0.44 (φ)
CD16 expression (mean MFI)	15.13	21.08	13.16	P = 0.0009 (φ)

A cohort of early RA patients (n = 73) were recruited for investigation. Baseline characteristics of the cohort is summarised in column [Entire cohort]. Patients with EULAR response data at week 14 post-initiation of therapy (n = 42) were furthermore split into non-responders [NR] or moderate/good responders [MOD/GOOD] and baseline characteristics in each subgroup analysed separately. Statistically significant differences between the two EULAR response subgroups [NR vs. MOD/GOOD] were examined. (‡) fishers statistical test (φ) mann-whitney U test.

#### Clinical predictors of response to steroid/methotrexate therapy

**Figure 5:**
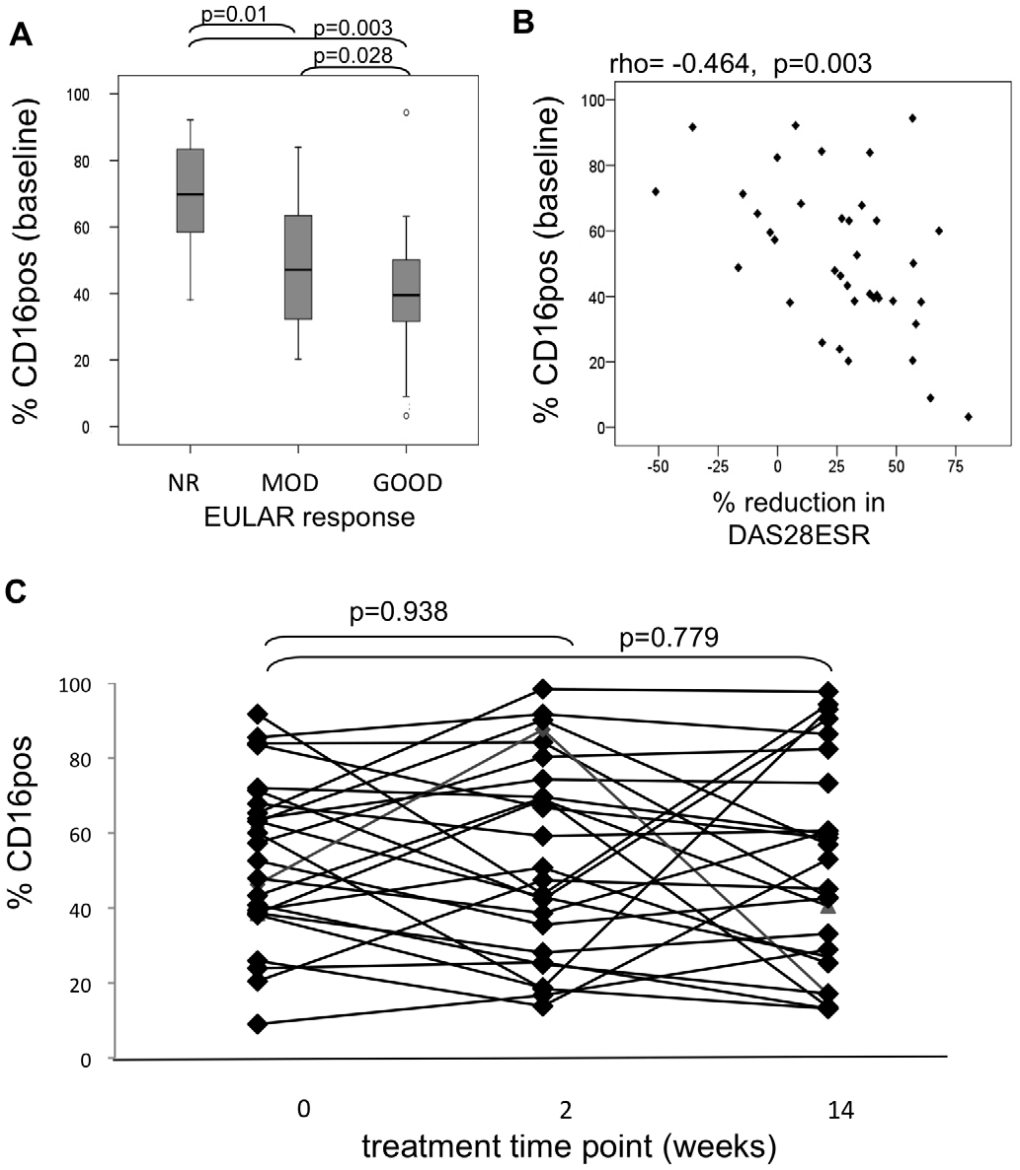
FcγRIIIa/CD16 expression on RA CD14^++^ monocytes pre- and post- methotrexate therapy. A) The percentage of CD16 positive CD14^++^ monocytes was measured at baseline (y-axis) and correlated with patient responses to methotrexate treatment. Patients were classified at week 14 post-initiation of therapy as non-responders (NR, n = 12), moderate (MOD, n = 12) or good (GOOD, n = 15) according to EULAR criteria and compared using Mann Whitney U test. Dots represent outliers. (B) The percentage reduction in DAS28-ESR between 0 and 14 weeks post-initiation of therapy (x-axis) and the % of CD14^++^ cells expressing CD16 at baseline (y-axis). Negative levels indicate increased DAS28ESR between baseline and week 14. Statistics; Spearman's correlation. C) The percentage of CD16 positive CD14^++^ monocytes was measured at baseline (wk0), 2 and 14 weeks after initiation of steroid/methotrexate treatment in early DMARD-naïve RA patients. Samples at 2 weeks mainly reflect influence of steroid treatment whereas week 14 samples will mainly reflect the effect of methotrexate therapy. Wilcoxon signed ranks test used to compare paired samples pre- and post- therapy.

Within this steroid/methotrexate treated cohort, baseline variable were examined for association with non- or mod/good- EULAR response to therapy by 14 weeks. There was no association between age, smoking status, presence of autoantibodies and baseline DAS28ESR with response to steroid/methotrexate therapy (Table 1). Significantly more women were non-responders to steroid/methotrexate therapy than men (p = 0.03, Table 1). The expression of CD16 on CD14^++^ monocytes prior to initiation of therapy in early-RA patients was found to be predictive of response to methotrexate; mean CD16 expression on CD14^++^ monocytes was significantly higher in non-responders compared to moderate and good responders (p = 0.0009, Table 1). Thus, the percentage of CD14^++^ monocytes expressing CD16 at baseline was significantly different between patients classified as EULAR non-responders and moderate or good responders at 14 weeks post-methotrexate therapy (Fig. 5A; p = 0.01 and p = 0.003 respectively). The percentage of CD16 positive CD14^++^ monocytes at baseline was also negatively correlated with the percentage reduction in DAS28-ESR levels between 0-14 weeks (Fig. 5B; spearman's rho = -0.464, p = 0.003). When the analysis was stratified by gender, CD16 was still significantly raised in EULAR non-responders compared to moderate and good responders (p = 0.0153) in female subjects. There were insufficient non-responders to assess this in male subjects. The levels of CD16 expression (MFI units, data not shown) and the percentage of CD14^++^ monocytes expressing CD16 were not significantly changed at 2 or 14 weeks post-treatment compared with baseline (p = 0.938 and p = 0.779 respectively; Wilcoxon signed ranks test Fig. 5C).

## Discussion

Monocytes are crucial players in the perpetuation of immune responses and joint damage in RA. The CD14^low^ monocyte subset has previously been the major focus of attention in RA due to reports of increased numbers in inflammatory diseases [28]–[31] and following reports suggesting they are the main produces of TNF in healthy controls [38]. However, in our current study we found no significant difference in the proportions of the CD14^low^ monocyte subset, consistent with another study in this area [32], or the level of FcγRIIIa/CD16 expression between RA and control subjects. In this study, we have demonstrated that the CD14^++^ monocyte subset in RA shows higher expression of FcγRIIIa/CD16 compared with healthy controls, as previously reported [6], [31], [35]–[37]. This increased expression may result in a cell that is more responsive to IgG-containing ICs with resultant cellular activation. FcγRIIIa/CD16 positive cells have been demonstrated to be the main producers of TNF in response to LPS [26]. Indeed, we have demonstrated that monocytes from RA patients show an enhanced capacity to produce TNF in response to IgG-containing ICs and the extent of TNF-production is correlated with the level of FcγRIIIa/CD16 expression on CD14^++^ monocytes. Therefore, we propose that higher FcγRIIIa/CD16 levels seen on CD14^++^ monocytes in RA may allow circulating ICs, found abundantly in RA patients, to provide an inflammatory drive toward the production of TNF and perpetuation of disease. Our data demonstrated higher TNF production in response to ICs in RA compared to healthy controls in contrast to recent work of Laurent *et al.* who reported TNF production in response to IC was similar between healthy controls and RA patients. The different results may be accounted for by a greater increase in FcγRIIIa/CD16 expression in RA relative to healthy controls in our study, which may accentuate the difference in TNF production between the groups. Furthermore, both studies used different experimental methodologies that may have directly impacted on the results, including method of monocyte isolation (unmanipulated PBMC vs. positive selection of monocytes), different ICs (HAG vs. ACPA-containing ICs derived from RA patients) and the measurement of intracellular TNF after 4 hours by flow cytometry compared with TNF release into the culture supernatant at 24 hours by ELISA. Various other studies have previously attempted to address this using a variety of ICs such as ACPA-containing [40], collagen-containing IC [41] and PEG-precipitated IC [42]. These studies were also unable to show any correlation between FcγRIIIa/CD16 and TNF production in response to the aforementioned IC, but once again different sources of monocytes/monocyte-derived macrophages were used (healthy controls) with a tendency for longer culture duration and the measurement of TNF release into culture supernatants, rather than intra-cellular TNF as employed in the current study. The extent to which these methodological differences directly impacted on the results from the different studies is currently unknown.

FcγR-mediated IC uptake, targeting into both MHC class I and II antigen presentation pathways and cross-presentation of IC-bound antigens has been demonstrated for FcγRIIIa/CD16 [46]–[49]. These CD14^++^/CD16^pos^ cells also co-express chemokine receptors and adhesion molecules thereby showing their capacity to migrate rapidly to sites of inflammation. Studies have demonstrated that magnetic-bead isolated CD16^pos^ monocytes adhere to activated endothelium and migrate into the joint more efficiently than CD16^neg^ monocytes due to increased adhesion molecule and chemokine receptor expression [50]. In RA, this CD16^pos^ phenotype of cells becomes expanded due to increased expression of CD16 on CD14^++^ monocytes and this cellular subset may be associated with increased recruitment into the joint. We have shown that monocytes from early-RA patients show intermediate levels of FcγRIIIa/CD16 expression between healthy controls and RA patients, thus increased FcγRIIIa/CD16 expression may become established as disease progresses. We have shown that steroid/methotrexate treatment does not down regulate FcγRIIIa/CD16 expression, even in patients achieving moderate/good clinical responses, consistent with previous longitudinal studies [35]. Although the work by Wijngaarden *et al.* 2005 showed a downregulation of CD16 after methotrexate therapy, this was a modest downregulation in the percentage of FcγRIIIa/CD16 positive cells and was not reproduced in MFI expression levels. We hypothesise that in the context of persistent autoantibody production in RA the CD14^++^/CD16^pos^ cellular subset may provide a continual drive to TNF-release and other effector functions, such as phagocytosis and antigen-presentation in the joint. Therefore, the presence of these monocytes may represent an important part of the pathophysiology of autoantibody-positive RA, which may be relatively resistant to methotrexate therapy and could be a potential therapeutic target.

We have demonstrated that increased FcγRIIIa/CD16 expression on CD14^++^ monocytes in RA may be important in determining non-response to methotrexate therapy. Expansion of this CD14^++^/CD16^pos^ subset may predict patients who will have persistent circulating IC-driven TNF production, irrespective of therapy, and as a consequence will demonstrate poorer responses and may need more aggressive treatment with anti-TNF/other biologics. FcγRIIIa/CD16 expression is not correlated with gender, DAS28-ESR score or the presence of systemic inflammation, suggesting that FcγRIIIa/CD16 may provide additional information above current measures related to response. However, this finding needs further validation, as several high FcγRIIIa/CD16 expressing patients demonstrated adequate responses to methotrexate therapy.

We propose that CD14^++^/CD16^pos^ monocytes warrant further investigation due to the potential pathogenic importance of the IC/FcγRIIIa activation pathway in driving inflammatory responses in RA. In addition, studies are needed to determine whether monocyte FcγRIIIa/CD16 expression levels could potentially be used as a prognostic or predictive biomarker of response to methotrexate therapy in RA. Furthermore, blockade of FcγRIIIa/CD16 represents an attractive target for future therapeutic intervention.

## Supporting Information

Figure S1 **Validation of non-specific blockade using immunoglobulin.** A) Expression levels of staining (MFI units) of IgG1-PE isotype control at increasing concentration of non-specific binding blockade using soluble immunoglobulin (IgG µg/ml). B) Expression levels of staining (MFI units) with CD16-PE antibody clone 3G8 with increasing concentrations of non-specific blocking using soluble immunoglobulin (IgG µg/ml) C) Comparison of mean staining levels (MFI units) of CD16-PE clone 3G8 on monocytes with or without immunoglobulin blockade (+/−IgG) (data from 5 RA patients and 3 healthy controls).
(TIFF)

Appendix S1 **Membership and Affiliations of the YEAR Consortium.**
(DOCX)
