# Supplementary figures and images for: FcγRIIIa Expression on Monocytes in Rheumatoid Arthritis: Role in Immune-Complex Stimulated TNF Production and Non-Response to Methotrexate Therapy
Source: PLoS One. 2012 Jan 3;7(1):e28918. doi: 10.1371/journal.pone.0028918 (PMC3250404; doi:10.1371/journal.pone.0028918)

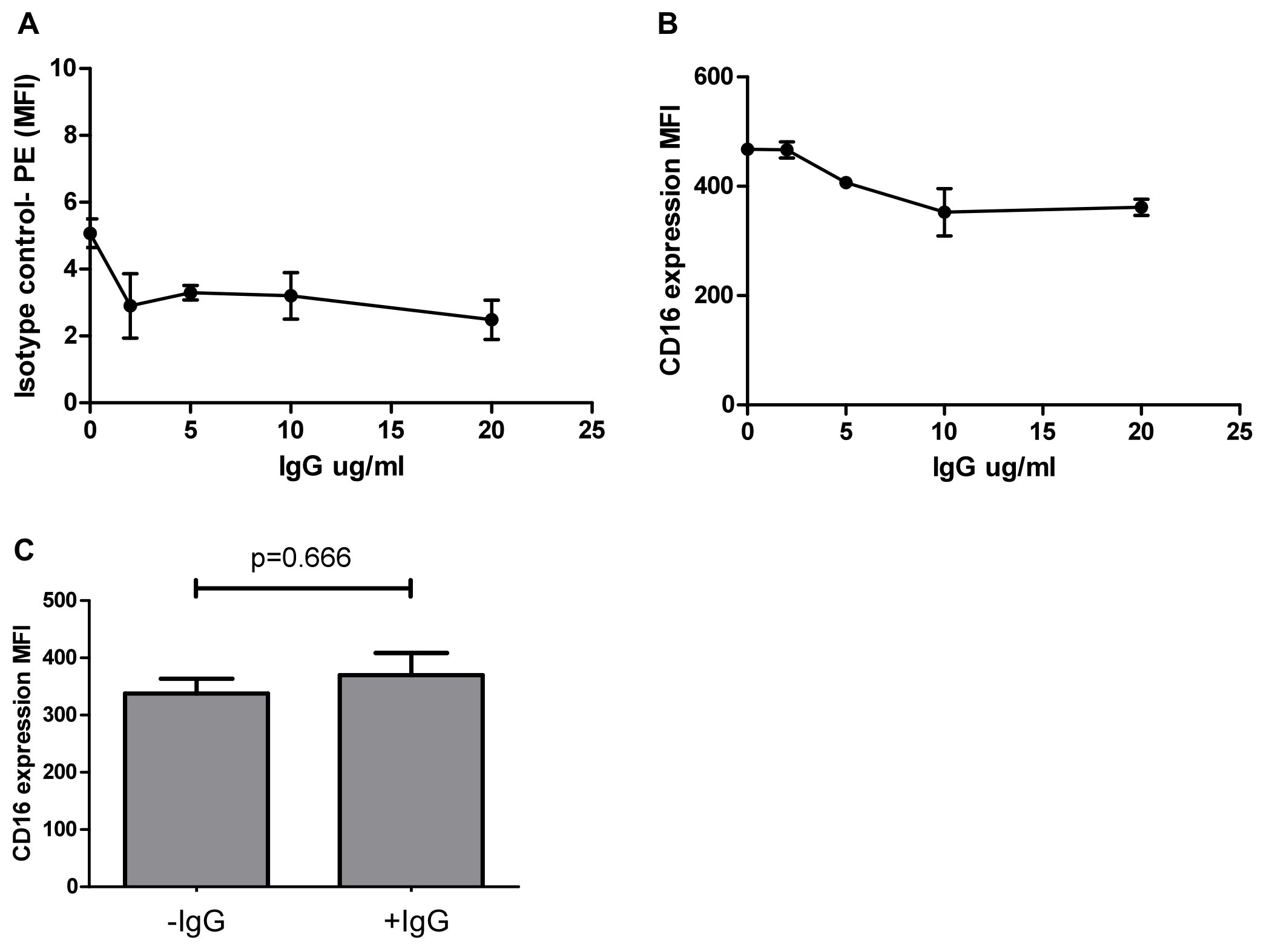

Supplement: Figure S1 — Validation of non-specific blockade using immunoglobulin. A) Expression levels of staining (MFI units) of IgG1-PE isotype control at increasing concentration of non-specific binding blockade using soluble immunoglobulin (IgG µg/ml). B) Expression levels of staining (MFI units) with CD16-PE antibody clone 3G8 with increasing concentrations of non-specific blocking using soluble immunoglobulin (IgG µg/ml) C) Comparison of mean staining levels (MFI units) of CD16-PE clone 3G8 on monocytes with or without immunoglobulin blockade (+/−IgG) (data from 5 RA patients and 3 healthy controls). (TIFF) [file pone.0028918.s001.tiff]
