# Supplementary material for: FcγRIIIa Expression on Monocytes in Rheumatoid Arthritis: Role in Immune-Complex Stimulated TNF Production and Non-Response to Methotrexate Therapy
Source: PLoS One. 2012 Jan 3;7(1):e28918. doi: 10.1371/journal.pone.0028918 (PMC3250404; doi:10.1371/journal.pone.0028918)
Supplement: Appendix S1 — Membership and Affiliations of the YEAR Consortium. (DOCX) [file pone.0028918.s002.docx]

**Management Team:** Paul Emery^1^, Philip Conaghan^1^, Ann W Morgan^1^, Mark Quinn^2^, Anne-Maree Keenan^1^, Elizabeth Hensor^1^, Julie Kitcheman^1^

**Consultants:** Andrew Gough^3^, Michael Green^2,3^, Richard Reece^4^, Lesley Hordon^5^, Philip Helliwell1^6^, Richard Melsom^6^, Sheelagh Doherty^7^, Ade Adebajo^8^, Andrew Harvey^9^, Steve Jarrett^9^, Gareth Huson^1^, Amanda Isdale^2^, Mike Martin^1^, Zunaid Karim^9^, Dennis McGonag1e^10^, Colin Pease^1^, Sally Cox^1^

**SpRs:** Victoria Bejarano^1^, Jackie Nam^1^

**Nurses:** Claire Brown^1^, Christine Thomas^1^, David Pickles^1^, Alison Hammond^1^, Beverley Neville^3^, Alan Fairclough^4^, Caroline Nunns^4^, Anne Gill^2^, Julie Green^2^, Belinda Rhys-Evans^1^, Barbara Padwell^1^, Julie Madden^10^, Lynda Taylor^10^, Sally Smith^1^, Heather King^1^, Jill Firth^6^, Jayne Heard^7^, Linda Sigsworth^6^

**Lab Staff:** Diane Corscadden^1^, Karen Henshaw^1^, Lubna-Haroon Rashid^1^, Stephen G Martin^1^, James I Robinson^1^

^1^Section of Musculoskeletal Disease, Leeds Institute of Molecular Medicine, University of Leeds, Chapel Allerton Hospital, Chapeltown Road, Leeds, LS7 4SA,UK

^2^York District Hospital, Wigginton Road, York, YO31 8HE, UK

^3^Harrogate District Hospital, Lancaster Park Rd, Harrogate, HG2 7SX, UK

^4^Huddersfield Royal Infirmary, Acre Street, Lindley, Huddersfield, HD3 3EA, UK

^5^Dewsbury District and General Hospital, Halifax Road Dewsbury, WF13 4HS, UK

^6^St Luke’s Hospital, Little Horton Lane, Bradford, BD5 0NA, UK

^7^Hull Royal Infirmary, Anlaby Road, Hull, HU3 2JZ, UK

^8^Barnsley District General Hospital, Gawber Road, Barnsley, S75 2PY, UK

^9^Pinderfields General Hospital, Aberford Road, Wakefield, WF1 4DG, UK

^10^Calderdale Royal Hospital, Salterhebble, Halifax, HX3 0PW, UK
